# Rape with Extreme Violence: The New Pathology in South Kivu, Democratic Republic of Congo

**DOI:** 10.1371/journal.pmed.1000204

**Published:** 2009-12-22

**Authors:** Denis Mukengere Mukwege, Cathy Nangini

**Affiliations:** 1 General Reference Hospital of Panzi, Bukavu, South Kivu, Democratic Republic of Congo; 2 Social Aid For the Elimination of Rape (SAFER), Toronto, Ontario, Canada; 2 Brain Research Unit, Low Temperature Laboratory, Helsinki University of Technology, Helsinki, Finland

## Abstract

Cathy Nangini and Denis Mukwege describe their work at the Panzi Hospital in the Democratic Republic of Congo, which treats women victims of rape with extreme violence that are often perpetrated at the hands of armed groups.

**Summary Points**

- In eastern Democratic Republic of Congo (DRC), the destructive and sadistic behaviour systematically perpetrated by different armed groups over the last ten years signals a new pathology we classify as rape with extreme violence (REV).
- REV is devastating to populations and can permanently damage women's reproductive capacity.
- The Panzi Hospital in Bukavu, South Kivu is one of the few established medical centres that has the capacity to treat REV cases.
- Current gaps in the provision of care for REV survivors include a lack of health care infrastructure, insufficient number of qualified psychotherapists, and challenges associated with socioeconomic reintegration.
- Trafficking of the DRC's minerals directly assists the occurrence of atrocities.

## Introduction

On any given night in eastern DRC, armed groups of men will overrun a village and divide into bands of three to five, forcing themselves into houses where they seize and serially rape women and young girls. Some mutilate female genitals with guns, pieces of glass, wood, or heated plastic. Some take their victims to the forest and torture them as sex slaves for days, months, or years.

As Stephen Lewis, the former UN special envoy for HIV/AIDS in Africa has said ([1, p. 32]), “the capacity for brutality by so many perpetrators – and on the flip side, the capacity for indifference by so many witnesses – is the ugly apex of a trend gone unchecked.”

This ugly apex has characterized the sexual violence landscape throughout history, across cultures, during times of conflict, and behind closed doors. It is in wartime, however, that rape becomes a coordinated, deliberate, en masse weapon of terror. Systematic rape is now understood as the defining tactic of modern conflicts [2]. The mass rape of hundreds of thousands of women have been reported in Europe and Nanking during the Second World War [3], Bangladesh [3], Rwanda [4], and the former Yugoslavia [4]. In both Rwanda and Yugoslavia, the International Criminal Tribunal has recognized rape as a means of ethnic cleansing and genocide [5],[6].

The ongoing violence in the eastern DRC is exemplary of the use of rape to not only terrorize, displace, and demoralize, but also to deliberately incur severe sexual trauma on a level that merits analysis in and of itself. This tactic has escalated over the past ten years in eastern DRC with impunity, garnering little international attention or response. The latest figures estimate 1,100 rapes per month as assessed between November 2008 to March 2009 [7]. One of us (DM) is a gynaecologist and obstetrician who has been treating sexual violence survivors in South Kivu for a decade and as such is a first-hand witness to the brutality and scale of this kind of rape. The purely destructive and sadistic behaviour perpetrated by different armed groups signals a new pathology in South Kivu that we classify as rape with extreme violence (REV).

In this article, we document the medical consequences of REV through the lens of the Panzi Hospital in Bukavu, South Kivu, one of the few established medical centres that has the capacity to treat REV cases. In the ongoing conflict of the DRC, much remains unknown and undocumented; we draw heavily on a few reports by nongovernmental organizations (NGOs) and other observers about the use and impact of rape in the DRC, and on our own extensive experience in the field.

## Rape with Extreme Violence

We consider rape an efficient form of biological warfare that is inexpensive to implement, effective over large areas, and does not particularly endanger the attackers. Its effectiveness relies on the perception, deeply embedded in patriarchal societies, that women's sexuality is a prefecture of male ownership, and it is linked to the persistence of unequal gender relations and particularly to the way women's bodies are regarded ([8], p. 45). Its impact is multiplied when the woman becomes pregnant and the attack is then passed on to the next generation [9].

Rape with extreme violence, as evidenced in the DRC today, is implemented in three ways: (i) gang rape, usually by three or more men, leading to a high risk of injury; (ii) genital mutilation; and (iii) intentional transmission of sexually transmitted diseases such as chlamydia and HIV. Extremely violent rape has been documented in the report of 492 REV survivors in South Kivu: 57.3% of the women were convinced that the extreme violence and cruelty inflicted on them by armed forces was “proof that there was a plan to destroy and exterminate the Congolese people or in any case the communities that these women belonged to” ([8], p. 48). Seventy-two percent of women reported that they were tortured during the rape itself (beaten, wounded with machetes, genitally mutilated/burned by drops of plastic melted by flame), and 12.4% had had objects inserted into their vaginas (sticks, bottles, green bananas, pestles coated in chili pepper, rifle barrels); some women, after being raped, were killed by shots fired into their vaginas ([8], p. 34). Many women interviewed also believed that the rapes are aimed at destroying women's identity by means of “ethnic cleansing,” and that spreading HIV/AIDS and impregnating women are also deliberate acts ([8], p. 49). Such a strategy was prevalent in the Rwandan genocide ([10], p. 49]).

## The Panzi Hospital

The Panzi Hospital is located in Bukavu, South Kivu, a city that has been relatively secure in recent years compared with surrounding areas in the forest and in North Kivu where there are direct military clashes. In terms of sexual violence treatment, it is the best-funded hospital in South Kivu, primarily receiving funds from Communauté des Eglises de Pentecote en Afrique Centrale (CEPAC), the largest democratically governed church network in the DRC, and external NGOs. The Panzi Hospital is a referral hospital, receiving the most severe REV cases from smaller centres, owing to its expertise in surgical repair of urogenital trauma, including urological-genital and rectal-genital fistulas, simple and diverse genital and/or anal wounds, diverse genital mutilation, and other complications such as fractures of the pelvis and femur.

Despite its support network and the overwhelming need for the services it provides, the Panzi Hospital continually faces a shortage of money, supplies, and resources to expand its base of qualified personnel. These shortfalls have, on occasion, impacted the hospital's ability to provide core medical services to sexual violence survivors. Currently the Panzi Hospital is running at maximum capacity, treating ten sexual violence survivors per day and devoting 200 of its 350 beds to these cases.

Sexual violence survivors are offered free treatment at the Panzi Hospital. Patients are first triaged, and those suffering from tuberculosis, malnutrition, or psychosis are identified. Screening for HIV, syphilis, and vaginal infections is provided, and if the rape has occurred within 72 hours, patients receive postexposure prophylaxis. In-hospital care is provided for those needing surgical treatment resulting from rape or childbirth trauma. Usual cases include urogenital fistula, enteric fistula, and trauma to the perineum.

Psychological screening and treatment are also provided by a welfare worker, social assistant, or psychologist, depending upon the severity of the trauma. As part of the spiritual therapy provided at the Panzi Hospital, the hospital chaplain helps in domestic reconciliation between husbands and wives. Husbands are counselled not to blame their wives for their traumas and the couple is counselled to forgive their aggressors to overcome their anger and bitterness.

From 1999 to August 2006, the Panzi Hospital treated 9,778 patients, of which 7,519 (76.9%) were confirmed as rape survivors. Table 1 shows the physical health profile of these patients. Three quarters of patients were in the 6- to 45-year age group. Attacks on women of child-bearing age severely impacts family life (to the extreme that many women are abandoned by their husbands) or the future prospect of one, brought on by the shame of rape and the associated psychological and physical consequences.

**Table 1:** Physical health profile of n = 7,519 sexual violence survivors treated at the Panzi Hospital (1999–Aug 2006).

Clinical Sequelae to Rape	Total	%
**Gynaecological problems**		
With	4,683	62.3
Without	2,836	37.7
**Sexually transmitted infections**		
Vaginal trichomonas	—	8.9
Genital infection	—	21.2
Urinary infection	—	18.2
Syphilis	—	3.0
HIV	—	4.5
Candida infection	—	14.1
No infection	—	30.1
**Surgeries performed**		
Fistula	1,225	29.8
Other	2,890	70.2

In an analysis of 836 raped women treated at the Panzi Hospital in 2006, the majority were married (56%) or widowed (21%); a sizeable fraction of women were abandoned by their husbands (11%). The majority of women were attacked at home (55%), in the field (19%), or in the forest (17%).

The Panzi Hospital is building its efforts to empower survivors who have nowhere to go after treatment and who often have limited skills. A number of programs are ongoing at the hospital, including: baking, soap works, sewing, embroidery, soy-milk/fruit juice making, tanning of leather goods, literacy, and microfinancing. Between 1999 and August 2006, 3,049 women have been enrolled in these programs.

## Impact of Rape

The war in eastern DRC has widowed a large number of women, forcing them to become heads of households without having had any preparation for this role. The war has destroyed women's means of production and, as a result, they live below the poverty line and many rely on food aid (when available) ([8], p. 25). Many women and girls have also been forced into survival sex, which makes them particularly vulnerable to sexual violence. An increase in domestic violence—a common fallout of war—has also been observed, as unemployment among men and uncertainty regarding the country's political future is on the rise ([8], p. 26).

REV has devastating consequences: it damages social cohesion and the identity of the rape survivor, which is exacerbated when rape is committed in public, searing shame into the collective memory of the community. The long-term outcomes translate into the slow death of a population incapable of reproducing. The impact of military rape on the population as a whole has far-reaching effects that ultimately undermine national, political, and cultural solidarity; it confuses the loyalties of all survivors and the identities of subsequent generations: “There is more than one way to commit genocide. One way is mass murder, killing individual members of a national, political or cultural group. Another is to destroy a group's identity by decimating cultural and social bonds. Martial rape does both.” ([11], p. 8).

## Gaps in the Provision of Care

It is tremendously important that access to medical care be established for as many rape survivors as possible. There are three main gaps in the provision of care: lack of health care infrastructure, an insufficient number of qualified psychotherapists, and the socioeconomic reintegration gap. A functioning health care system needs to be established to define health territories or areas that can effectively manage sexual violence cases. Such cases can be identified and treated at this level, and, if necessary, the decision to transfer patients to a superior facility can be made. In this way, the access to health care would become more evenly distributed across the province, and would, additionally, alleviate the incoming patient load on the Panzi Hospital, which treats the most severe cases. The Panzi Hospital could participate in the training of personnel and play an advisory role in the system; however, money and resources remain the biggest obstacles in establishing such a network. Medical care, while essential, is but the first milestone to be reached for a holistic recovery. Psychosocial treatment is essential to help re-establish dignity and the desire to live. However, the sheer volume of rape has created an enormous gap in psychosocial care in a region where the number of qualified psychotherapists is very limited. Given the large number of survivors and the long period of time required for healing, it is necessary to train social workers in each health region and help them get settled for the long term to allow localized provision of care.

Of approximately 3,600 women treated per year at the Panzi Hospital, about one-third have sufficient economic support to allow them to recover their dignity and place in society. Some provision of social-economic care has been provided at the Panzi Hospital since last year, including programs for trade apprenticeship, management of microfinance grants, informal education, and literacy teaching. Daycare is also provided for schoolgirls who have children as a result of rape.

## Gaps in Information

The Panzi Hospital is one of the few establishments in the DRC maintaining detailed records of sexual violence reports. This is important not only for characterizing and assessing the consequences of the crisis, but also for understanding the underlying factors that fuel REV.

**Figure 1:**
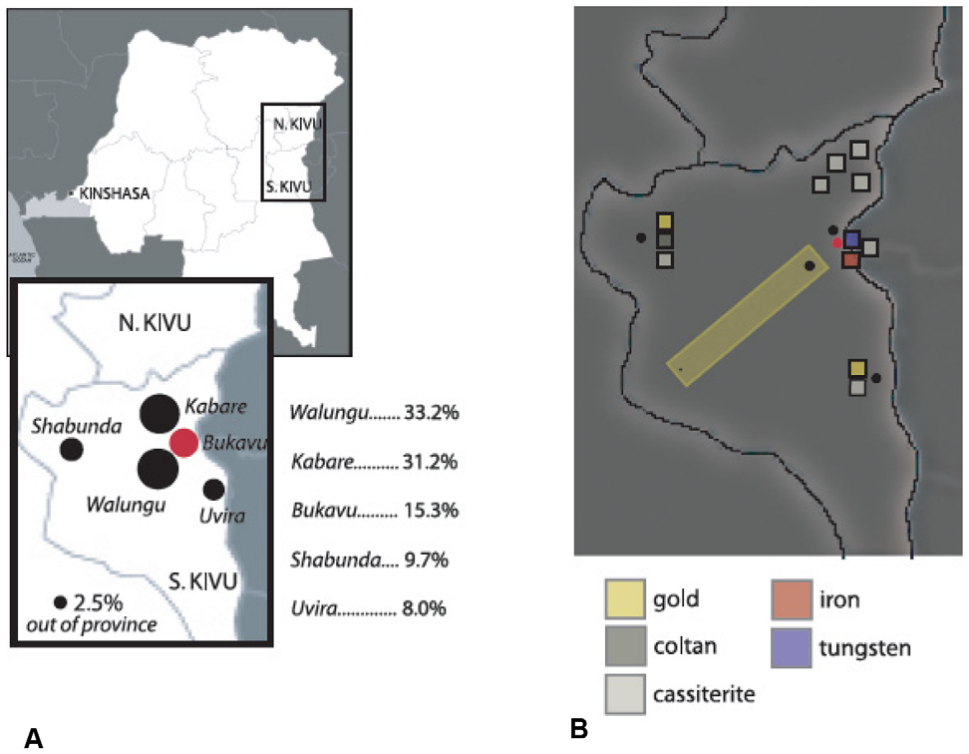
Map of South Kivu showing areas of documented rape and mineral wealth. (A) Regional breakdown of rape location of sexual violence survivors who received treatment at the Panzi Hospital (1999-Aug 2006). The Bukavu area, where the Panzi Hospital is located, is shown in red. (B) Known mineral deposits near the five regions (black dots) identified in the left panel. Sources: [15]–[18].

In 2002, a UN Panel of Experts provided extensive documentation supporting the link between economic exploitation and human rights abuses, revealing that various armed groups have built up a self-financing war economy centred on mineral exploitation ([12], p. 5) (Box 1). The Panel also determined that companies and individuals operating in the DRC who, directly or indirectly, contribute to the revenues of the “elite network”—a small core of political and military/rebel elites and business persons involved in exploitation activities ([12], p. 6)—“contribute to the ongoing conflict and to human rights abuses” ([12], p. 32). We examined whether Panzi Hospital data support the link between sexual violence and mineral wealth, and we compared regions in South Kivu characterized by REV with their known mineral resources. Figure 1 shows the regional breakdown of rape location from Panzi Hospital records (1999–Aug 2006) and corresponding mineral assets compiled from publicly available sources. The area of the circles is proportional to the fraction of the hospital's survivors attacked in these regions. The areas are centred around: Walungu (3,251 [33.2%]), Kabare (3,050 [31.2%]), Bukavu (1499 [15.3%]), Shabunda (951 [9.7%]), and Uvira (785 [8.0%]). A small proportion (242 [2.5%]) of cases treated at the Panzi Hospital occurred outside of South Kivu. Since data regarding the mode of transport to the hospital were not available, the map has not been corrected for transport bias.

**Box 1. Mineral Wealth in Regions Where REV Is Rampant**

Publicly available data indicate that the five foci of REV in South Kivu (Figure 1) are rich in mineral resources and under the control of armed forces.

The Walungu region is rich in gold and cassiterite (a tin oxide mineral), and is one of the administrative territories of the FDLR (Democratic Liberation Forces of Rwanda), along with Kabare, Shabunda, and Uvira [19]; the FARDC (Armed Forces of the Democratic Republic of Congo) have also been documented as being actively involved in gold and cassiterite mining in the Walungu area [15]. A 210-km transect (the Twangiza-Namoya gold belt, gold box in Figure 1B), running southwest from Bukavu through the Walungu area, contains large gold deposits [17].

Kabare (Nindja) has deposits of coltan (which contains tantalum, widely used in electronics manufacture). The FDLR [19] and several other militias have been implicated in the control of Kabare mines [20], and Uvira is close to gold and cassiterite reserves [16].

Shabunda is a small mining town extremely rich in mineral resources, especially gold, coltan, and cassiterite, and is surrounded by jungle. It has been largely held by Rwandan forces and was heavily besieged by Mai-Mai militiamen who kidnapped townswomen for use as sex slaves. In 2002, the Guardian reported 2,000 women still missing [21]. Rebel forces have set up their own mining company in Shabunda (Great Lakes Mining Company), which monopolizes the exploitation of natural resources [22].

The correlation between sexual violence and mineral wealth needs to be consolidated with more data and further analysis, and is crucial for informing better REV response and prevention strategies.

## Gaps in International Response

Despite the legal structures in place recognizing rape as a crime against humanity and a war crime [13], sexual violence remains low on the international radar. While mass rape in the eastern DRC continues to be documented widely by NGOs and the UN, this has yet to curb the use of REV and end impunity for such crimes against humanity.

Part of the indifference may be a result of misrepresentation of the conflict in DRC as a “civil war.” In fact, the Congo conflicts have involved the armies of seven African states, and criminal groups from the armies of Rwanda, Uganda, Zimbabwe, and the DRC have benefited by building up “a self-financing war economy centred on mineral exploitation” ([12], p. 5]). Violence is indeed carried out by “warlords and drug-crazed child soldiers” [14], but the driving mechanism behind the war is a rational system that ultimately benefits the military, commercial, and political elites from the DRC and other countries [12]. Finding the political will to restrain the activity of the elite networks and their supporters is “the most important element in effectively halting the illegal exploitation of resources” [12], p. 28]. Only then can the new pathology of rape with extreme violence be eradicated. Our recommendations for addressing REV in DRC are contained in Box 2.

**Box 2. Recommendations for Addressing REV in DRC**

We make the following recommendations in response to five key factors that contribute to the widespread deployment of REV:

1. Ensure that leaders in the eastern DRC and neighbouring regions take responsibility and act to end the atrocities in DRC.
2. Help create and train a national army and police force that are ready and able to protect civilians.
3. Develop a mechanism for the traceability of minerals as a way forward for ultimately reducing the reliance of armed groups on DRC's resources.
4. Address impunity for sexual violence in the International Criminal Court (ICC) and the current national courts, including the period before the creation of the ICC.
5. Assist all organizations that provide psychological, medical, legal, and social care to REV survivors.

## Supporting Information

Alternative Language Summary S1 French translation of the summary points by Dr. Olivier Couture.
(0.03 MB DOC)

## Abbreviations

CEPAC: Communauté des Eglises de Pentecote en Afrique Centrale
DRC: Democratic Republic of Congo
NGO, nongovernmental organization; REV: rape with extreme violence
