# Supplementary material for: Rape with Extreme Violence: The New Pathology in South Kivu, Democratic Republic of Congo
Source: PLoS Med. 2009 Dec 22;6(12):e1000204. doi: 10.1371/journal.pmed.1000204 (PMC2791171; doi:10.1371/journal.pmed.1000204)
Supplement: Alternative Language Summary S1 — French translation of the summary points by Dr. Olivier Couture. (0.03 MB DOC) [file pmed.1000204.s001.doc]

French translation of Summary Points of **Rape with Extreme Violence: The New Pathology in South Kivu, Democratic Republic of Congo**

Denis Mukwege^1^, Cathy Nangini^2^*

- - Les actions destructives et sadiques perpétrées par les différentes groupes armés dans l'est de la République Démocratique du Congo (RDC) nous obligent à définir une nouvelle pathologie: le viol avec violence extrême (VVE).
  - Le VVE a un effet dévastateur sur les populations et endommage irréversiblement le système reproducteur des femmes
  - L'hôpital Panzi à Bukavu au sud-Kivu est un des seuls centres médicaux qui ont la capacité de traiter les cas de VVE.
  - Le traitement des victimes de VVE comporte toujours des lacunes, en particulier un manque d'infrastructures médicales et un nombre insuffisant de psychothérapeutes qualifiés, sans compter les défis associés à la réintégration socioéconomique.
  - Le trafic des ressources minières de la RDC contribue directement à la perpétration de ces atrocités
